# PI-RADS 2.1 und strukturierte Befundung der Magnetresonanztomographie der Prostata

**DOI:** 10.1007/s00117-021-00868-6

**Published:** 2021-07-02

**Authors:** Andreas Hötker, Olivio F. Donati

**Affiliations:** Institut für Diagnostische und Interventionelle Radiologie, Universitätsspital Zürich, Rämistrasse 100, 8091 Zürich, Schweiz

**Keywords:** Prostatakarzinom, Multiparametrische Magnetresonanztomographie, Prostataspezifisches Antigen, Transitionalzone, Scoringsystem, Prostatic neoplasms, Multiparametric magnetic resonance imaging, Prostate-specific antigen, Transitional zone, Scoring system

## Abstract

**Klinisches/methodisches Problem:**

Die Identifikation klinisch signifikanter Prostatakarzinome bei gleichzeitigem Vermeiden einer Überdiagnostik niedrigmaligner Tumoren stellt eine Herausforderung in der klinischen Routine dar.

**Radiologische Standardverfahren:**

Die gemäß PI-RADS-Richtlinien (Prostate Imaging Reporting and Data System Guidelines) akquirierte und interpretierte multiparametrische Magnetresonanztomographie (MRT) der Prostata ist als klinischer Standard bei Urologen und Radiologen akzeptiert.

**Methodische Innovationen:**

Die PI-RADS-Richtlinien sind neu auf Version 2.1 aktualisiert worden und beinhalten neben präzisierten technischen Anforderungen einzelne Änderungen in der Läsionsbewertung.

**Leistungsfähigkeit:**

Die PI-RADS-Richtlinien haben entscheidende Bedeutung in der Standardisierung der multiparametrischen MRT der Prostata erlangt und bieten Vorlagen zur strukturierten Befundung, was die Kommunikation mit dem Zuweiser erleichtert.

**Bewertung:**

Die nun auf Version 2.1 aktualisierten Richtlinien stellen eine Verfeinerung der verbreiteten Version 2.0 dar. Dabei wurden viele Aspekte der Befundung präzisiert, einige vorbekannte Limitationen bleiben jedoch bestehen und erfordern die weitere Verbesserung der Richtlinien in kommenden Versionen.

## Abstract (en)

**Clinical/methodological issue:**

The detection of clinically significant prostate cancers while simultaneously avoiding over-diagnosing tumors with low malignant potential is a challenge in clinical practice.

**Standard radiological methods:**

Multiparametric prostate magnetic resonance imaging (MRI) in accordance with the Prostate Imaging Reporting and Data System (PI-RADS) guidelines is accepted as standard-of-care with both urologists and radiologists.

**Methodological innovations:**

The PI-RADS guidelines have been updated to version 2.1, including revised technical recommendations and changes to the scoring of lesions.

**Performance:**

The PI-RADS guidelines have had great impact on the standardization of multiparametric prostate MRI and offer templates for structured reporting. This simplifies communication with the referring physician.

**Achievements:**

The new version 2.1 of the guidelines represents an evolutionary improvement of the widely accepted version 2.0. Several aspects of reporting have been revised—however, some pre-known limitations persist, which will require further refinement in the future.

Die multiparametrische Magnetresonanztomographie (mpMRT) der Prostata zur Detektion von suspekten Herden ist in der Abklärung von Patienten mit Verdacht auf Prostatakarzinom in den letzten Jahren zum klinischen Standard geworden [3]. Die bisher gültige Guideline PI-RADS 2.0 [35] wurde 2019 einer Überarbeitung unterzogen, um zwischenzeitlich bekannt gewordene Unklarheiten auszuräumen. Die dabei entstandene und hier vorgestellte PI-RADS-Richtlinie in der Version 2.1 stellt somit die aktuell gültige Grundlage für die Befundung der mpMRT dar [32].

## Entstehung und Bedeutung von PI-RADS

In der Prostatadiagnostik steht die Detektion von klinisch signifikanten Tumoren im Vordergrund, also Prostatakarzinomen, die nach PI-RADS 2.0 einen ISUP-Grad ≥ 2, eine Tumorgröße ≥ 0,5 ml und/oder eine extraprostatische Ausbreitung zeigen und von denen anzunehmen ist, dass sie einen Einfluss auf das weitere Überleben des Patienten haben werden [35]. In mehreren großen, prospektiven klinischen Studien konnte der Wert einer präbioptisch durchgeführten MRT belegt werden. Diese führt zu einer höheren Detektionsrate klinisch signifikanter Tumoren – ohne jedoch die Zahl der entdeckten niedrig-malignen Tumoren zu erhöhen. Außerdem führt eine präbioptisch durchgeführte mpMRT der Prostata zu einer geringeren Anzahl notwendiger Biopsiestanzen [1, 15, 30]. Diese Vorteile der gezielten Prostatabiopsie, für welche die Visualisierung suspekter Herde mittels mpMRT eine *Conditio sine qua non* darstellt, führten zur Aufnahme der mpMRT der Prostata in die deutschen, europäischen und amerikanischen urologischen Leitlinien [2, 5, 20].

Die 2012 eingeführten und 2015 und 2019 überarbeiteten PI-RADS-Richtlinien zur Akquisition, Interpretation und Befundung trugen wesentlich zur Akzeptanz und zum Erfolg der mpMRT der Prostata bei und wurden sowohl durch radiologische als auch urologische Experten validiert [33].

Die letzte Iteration der Guidelines fand 2019 mit der Aktualisierung der im Jahr 2016 publizierten Version PI-RADS 2.0 [35] auf Version 2.1 [32] statt. Neben der Klarstellung einzelner technischer Anforderungen an die Untersuchungstechnik sollte die neue Version primär Inkonsistenzen bei der Vergabe der Scores reduzieren und damit die Reproduzierbarkeit der Befundung verbessern. Gleichzeitig sollte damit eine Reduktion der klinisch schwierig zu handhabenden *indeterminierten* Läsionen erreicht werden [22].

Der vorliegende Artikel stellt die wichtigsten Änderungen durch PI-RADS 2.1 und die bisher veröffentlichte Literatur zur Validierung dieses Scoringsystems vor und beleuchtet noch offene Fragen und Probleme. Für Informationen zur grundsätzlichen Befundung multiparametrischer Prostata-MRT wird auf bereits in diesem Journal veröffentlichte Übersichtsarbeiten verwiesen [10, 27].

## PI-RADS 2.1 Guidelines

Im Vergleich zu den mit dem Wechsel von Version 1.0 auf Version 2.0 verbundenen Änderungen sind die Anpassungen in Version 2.1 deutlich geringer und betreffen neben einzelnen Veränderungen bzgl. Empfehlungen zur technischen Durchführung primär Präzisierungen in der Charakterisierung einzelner Läsionen insbesondere in der Transitionalzone (TZ), der zentralen Zone (CZ) und im anterioren fibromuskulären Stroma (AFMS). Das grundsätzliche Vorgehen in der Befundung einer mpMRT bleibt unverändert, und auch die für die jeweilige Zone als dominant angesehene Sequenz bleibt erhalten. Vorerst wird auch weiterhin eine mpMRT mit T2-gewichteten, diffusionsgewichteten (DWI) und kontrastmittelverstärkten Sequenzen (DCE) empfohlen, wobei aufgrund der zunehmenden Zahl von Untersuchungen gerade unter Active Surveillance auch ein biparametrischer Ansatz ohne Kontrastmittelgabe zumindest in spezialisierten Zentren an Zuspruch gewinnt.

### Technische Anpassungen in der Bildakquisition

In PI-RADS 2.1 wurden einzelne Klarstellungen und Modifikationen der empfohlenen technischen Minimalanforderungen bei der Untersuchungsdurchführung vorgenommen.

- Typischerweise werden zur Untersuchung der Prostata hochaufgelöste T2-gewichtete Turbo-Spin-Echo-Sequenzen in axialer (entweder gekippt entlang der Prostatalängsachse oder streng axial), koronarer und sagittaler Schnittführung akquiriert. In Version 2.1 wurde präzisiert, dass zumindest eine axiale und eine weitere sagittale oder koronare Sequenz aufgenommen werden muss. Gerade zur Beurteilung von Läsionen der Transitionalzone bleibt die Akquisition von T2-gewichteten Sequenzen in allen 3 Ebenen aber weiterhin empfehlenswert.
- In PI-RADS 2.0 wurde für die Akquisition der DWI-Sequenzen die Verwendung eines niedrigen b‑Werts > 0 s/mm^2^ (z. B. 50–100 s/mm^2^) präferiert, um den Einfluss von Perfusionseffekten bei niedrigen b‑Werten zu minimieren. In Version 2.1 wurde dies weiter gefasst, und es wird nun die Akquisition eines niedrigen b‑Werts von 0–100 s/mm^2^ und eines mittleren b‑Werts von 800–1000 s/mm^2^ empfohlen. Der für die Auswertung wichtige hohe b‑Wert (≥ 1400 s/mm^2^) soll entweder separat akquiriert oder aus den vorangegangenen Werten über lineare Interpolation berechnet werden (sog. berechneter oder virtueller hoher b‑Wert).
- Bezüglich DCE-Sequenzen wurden in PIRADS 2.1 die technischen Anforderungen reduziert, und es wird nun eine zeitliche Auflösung ≤ 15 s (früher ≤ 10 s, bevorzugt < 7 s) und die Verwendung von 3‑D-Gradientenechosequenzen präferiert – beides erlaubt eine höhere örtliche Auflösung und damit bessere Abgrenzbarkeit von Läsionen. Die DCE-Sequenz soll dabei einen Zeitraum von mindestens 2 min nach Kontrastmittelgabe abdecken.

### Änderungen in den Befundkriterien für fokale Läsionen

Zur Verbesserung der Reproduzierbarkeit zwischen verschiedenen Befunder*innen wurden in der neuen Version der PI-RADS-Richtlinie einzelne Formulierungen präzisiert. Gleichzeitig erfolgt erstmals die Aufnahme von Läsionen der zentralen Zone und des anterioren fibromuskulären Stromas, allerdings noch ohne spezifische Bewertungskriterien.

**Figure 1:**
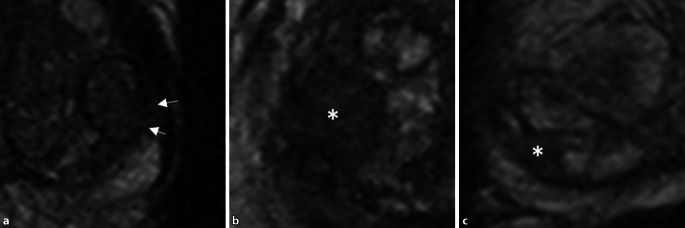

**Figure 2:**
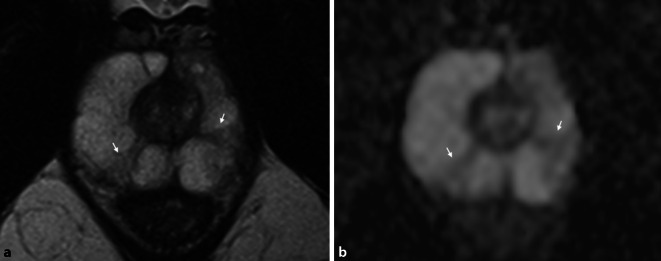

**Figure 3:**
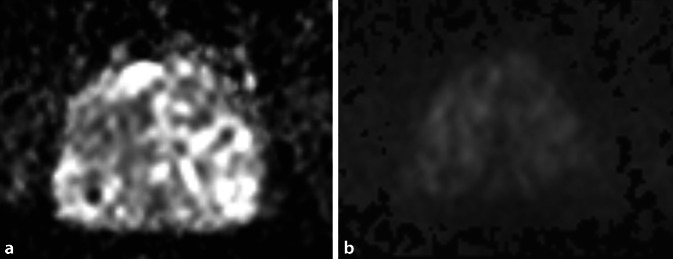

- In der Transitionalzone sollen grundsätzlich alle fokalen Läsionen einen Score erhalten, die entweder direkte Kriterien der Malignität oder ein von der Umgebung deutlich abweichendes Signalverhalten zeigen. Hier werden neuerdings alle typischen Knoten einer benignen Prostatahyperplasie (BPH) der PIRADS-Kategorie 1 (vormals 2) zugeteilt und sollten nicht mehr separat ausgewiesen werden. Unter atypische BPH-Knoten mit einem PI-RADS-Score von 2 fallen nun ein fast vollständig scharf berandeter Knoten, ein homogener, umschriebener Knoten ohne scharfe T2w-hypointense Grenze und ein homogenes, geringgradig T2w-hypointenses Areal zwischen 2 Knoten (Abb. 1). Diese Läsionen können bei deutlicher Diffusionsrestriktion (DWI-Score ≥ 4) jetzt einen PI-RADS-Score von 3 erhalten. Die mögliche Aufwertung von PI-RADS-3-Läsionen (auf T2-gewichteten Sequenzen) in der Transitionalzone bei einem DWI-Score von 5 zu einem Gesamtscore von 4 bleibt unverändert bestehen.
- In der Beurteilung von Läsionen in der Diffusionsbildgebung sowohl in der peripheren Zone (PZ) als auch in der TZ wurden ebenfalls einzelne Unklarheiten ausgeräumt: Lineare oder keilförmige Läsionen, die hypointens im ADC und/oder hyperintens im hohen b‑Wert-Bild sind, erhalten einen Score von 2 (Abb. 2). Fokale Hypointensitäten im ADC und/oder fokale Hyperintensitäten im hohen b‑Wert-Bild (solange sie noch nicht als „markedly diffusion-restricted“ bewertet werden), sowie Läsionen, die ausgeprägt hypointens im ADC *oder* ausgeprägt hyperintens im hohen b‑Wert-Bild sind (Abb. 3), werden alle mit einem DWI-Score von 3 versehen.
- Bezüglich der Beurteilung der DCE-Sequenzen wurde die Aussage zum negativen Kontrastmittelverhalten ergänzt. Als *negativ* werden nun alle Läsionen bezeichnet, die entweder keine frühzeitige Kontrastmittelaufnahme oder eine diffuse multifokale Kontrastmittelaufnahme zeigen, die nicht mit einer fokalen Läsion auf den übrigen Sequenzen korrespondiert.
- Die CZ erstreckt sich symmetrisch auf beiden Seiten von den Samenleitern an der Prostatabasis bis zum Verumontanum und lässt sich auf koronaren T2-gewichteten Sequenzen am besten abgrenzen. Tumoren der CZ sind sehr selten – meist erfolgt eine Infiltration der zentralen Zone durch Tumoren, die im angrenzenden Gewebe entstanden sind. Da das Gewebesignal der CZ auch bei Gesunden in T2w und ADC hypointens ist, lassen sich Tumoren in diesem Bereich aber oftmals schwer erkennen. Als diagnostisches Kriterium gilt eine Asymmetrie in den T2-gewichteten Sequenzen, in der Diffusionsbildgebung oder nach Kontrastmittelgabe. Es existieren allerdings auch Normvarianten mit Asymmetrie zu einer Seite, was die Diagnostik erschwert.
- Das anteriore fibromuskuläre Stroma kann ebenfalls durch angrenzende Tumoren insbesondere in der Transitionalzone infiltriert werden – hier gilt, dass sich die Befundung an der Zone des vermuteten Ursprungs des Tumors orientiert.
- Bezüglich der Berechnung des Prostatavolumens wurde präzisiert, dass die anterior-posteriore und longitudinale Ausdehnung auf einer sagittalen T2-gewichteten Sequenz, die transversale Ausdehnung auf einer axialen T2-gewichteten Sequenz ermittelt werden sollen.
- Auf der Sektorenkarte, die bisher 36 Zonen für die Prostata, zwei für die Samenblasen und eine für die membranöse Urethra enthielt, wurden zwei neue Zonen in der basalen medialen Prostata hinzugefügt.

## Strukturierte Befundung

Die strukturierte Befundung der Prostata-MRT zählt zu den wichtigsten Möglichkeiten zur Verbesserung der Befundkonsistenz und -vergleichbarkeit und erleichtert die Kommunikation mit den zuweisenden Ärzten [28, 36]. Die Version 2.1 der PI-RADS Guidelines [32] enthält im Appendix eine strukturierte Befundvorlage, die in dieser oder gering abgewandelter Form in den meisten Zentren zum Einsatz kommt. Die folgenden Punkte sind dabei von besonderer Bedeutung und sollten Teil des strukturierten Befundes sein:

- *Klinische Informationen und Indikation*: Letzter PSA-Wert, vorangegangene Biopsien oder Therapien (Bestrahlung, Androgendeprivationstherapie), auffällige digitale rektale Untersuchung etc.
- *Technik*: Aussage zur PI-RADS-Kompatibilität, Feldstärke, Spulenauswahl, Kontrastmittelgabe und Sequenzauswahl
- *Prostata*:
  - Größe (L × B × H) und Volumen, PSA-Dichte
  - Aussage zur Bildqualität
  - Hämorrhagische Prostataareale
- *Läsionen*: Jede Läsion separat (max. 4 Läsionen), mit der Läsion mit dem höchsten PI-RADS Score beginnend
  - Ort (Sektorenkarte und ggf. Serien‑/Bildnummer)
  - Größe der Läsion
  - Signalverhalten in T2w, DWI und DCE
  - Kontakt zum Prostatarand
- Staging/übriges Becken
  - Extraprostatische Ausbreitung
  - Distanz zu oder Infiltration des neurovaskulären Bündels (für PI-RADS 4/5)
  - Infiltration der Samenblasen
  - Lymphknoten- oder Knochenmetastasen
- Gesamtbeurteilung

**Figure 4:**
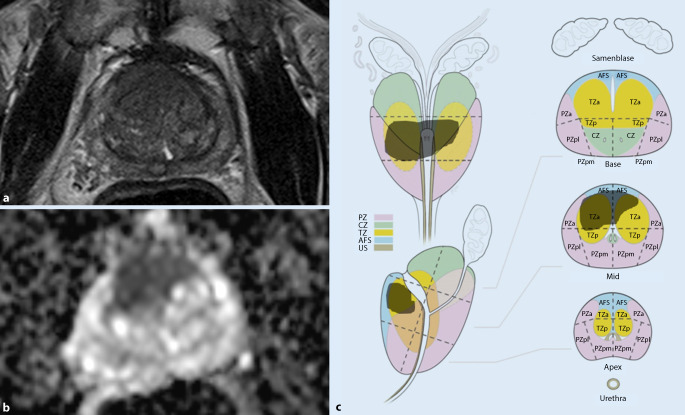

Für die verbliebenen Freitextfelder bieten die PI-RADS 2.1-Guidelines ein eigenes Stichwortverzeichnis an, so dass auch dort die Variabilität zwischen einzelnen Befunder*innen möglichst geringgehalten werden soll. In viele Befundvorlagen ist gleichzeitig auch die Sektorenkarte der Prostata nach Version 2.1 integriert – die Markierung der Läsionen erleichtert den biopsierenden Kolleg*innen die korrekte Identifikation der beschriebenen Läsionen (Abb. 4). Zur Unterstützung der Befunderstellung stehen mittlerweile mehrere Softwareprodukte verschiedener Hersteller zur Verfügung, welche die einfache und grafisch ansprechende Erstellung eines strukturierten Befundes erleichtern.

### Bisherige Validierung der Änderungen durch PI-RADS 2.1

Die bisherigen Studienergebnisse zum Einfluss der Änderungen in PI-RADS 2.1 gegenüber 2.0 auf die Genauigkeit in der Detektion von klinisch signifikanten Karzinomen und die Reproduzierbarkeit zwischen verschiedenen Befunder*innen sind teils widersprüchlich. Während einige Studien zumindest für Läsionen der Transitionalzone von einer gering erhöhten Genauigkeit oder einer gering verbesserten Reproduzierbarkeit berichten [4, 6, 16, 31, 34, 37], zeigen andere Studien praktisch keinen Unterschied in der Tumordetektion zwischen den beiden Scoringsystemen [7, 14, 17, 26]. Dies liegt vermutlich an der geringen Zahl von Läsionen der Transitionalzone, die in der klinischen Routine durch Version 2.1 eine Aufwertung erfahren – während die überwältigende Mehrzahl insbesondere der Läsionen mit klinisch signifikanten Karzinomen bereits in Version 2.0 korrekt kategorisiert wurde. Daher wird der Einfluss der neuen Version 2.1 auf das klinische Management von Patienten bisher als gering angesehen [17], wobei wahrscheinlich erst größere prospektive Studien diesbezüglich eine endgültige Aussage ermöglichen. Auch die zur Klassifizierung von Läsionen in der peripheren Zone vorgenommenen Änderungen (bspw. die Definition linearer Diffusionsrestriktionen als PI-RADS 2) scheint nur sehr selten ein „downgrading“ von PI-RADS 3 zu PI-RADS 2 herbeizuführen [17]. Die Abschätzung des Prostatavolumens scheint in PI-RADS 2.0 geringfügig genauer zu gelingen, als in Version 2.1 [11].

## Limitationen der aktuellen PI-RADS-Richtlinien

Die Version 2.1 der PI-RADS-Guidelines versteht sich als Verfeinerung der bestehenden Guidelines in Version 2.0. Die vorgenommenen Änderungen fallen demnach auch vergleichsweise gering aus, und während einzelne Kritikpunkte und Inkonsistenzen vorangegangener Versionen bereinigt wurden, bleiben bekannte Limitationen von PI-RADS bestehen.

Obwohl die strukturierte Befundvorlage die Angabe des letzten PSA-Werts und ggf. der PSA-Dichte verlangt, haben klinische Informationen aktuell keinen Einfluss auf die Befundung bzw. die Zuweisung von Scores zu Läsionen. Einzelne Publikationen berichten allerdings, dass sich z. B. unter Einbeziehung der PSA-Dichte eine Verbesserung der Genauigkeit [18, 24] oder personalisierte Entscheidungen hinsichtlich der Notwendigkeit einer Biopsie [8] erreichen ließen. Die PI-RADS-Richtlinien enthalten bisher auch keine Empfehlungen für die weitere Therapie bzw. Diagnostik der Patienten (im Unterschied z. B. zu den Richtlinien für die Mammadiagnostik, BI-RADS). Allerdings sind hier bereits Vorschläge des PI-RADS Steering Commitees zur Implementation der MRT in die Abklärung von Patienten mit Verdacht auf Prostatakarzinom veröffentlich worden [21], so dass eine Inkorporation in zukünftige Versionen wahrscheinlich ist.

In den meisten Zentren werden neben PI-RADS-4- bzw. -5-Läsionen auch *indeterminierte* Läsionen mit einem Score von 3 biopsiert, um kein klinisch signifikantes Karzinom zu verpassen. Dies liegt auch an der schwierigen Beurteilung dieser PI-RADS-3-Läsionen begründet, wobei die Änderungen in PI-RADS 2.1 insbesondere für Läsionen in der Transitionalzone nach bisheriger Studienlage keinen großen Effekt auf das klinische Management zeigen [17]. Hier könnte die Einbeziehung quantitativer Parameter (z. B. des ADC-Werts) prinzipiell hilfreich sein und neben einer besseren Detektion von höhergradigen Tumoren zusätzlich eine Reduktion der Variabilität zwischen den einzelnen Befunder*innen ermöglichen. Hierfür ist aber eine Standardisierung der Messungen zwischen verschiedenen Institutionen und Geräten unerlässlich [9, 29]. Für die Schwelle von 1,5 cm zwischen einer Läsion mit einem Score von 4 vs. 5 sind nur wenig Daten vorhanden. Auch diese Entscheidung könnte durch weitere quantitative Parameter verbessert werden [25]. Der potenzielle Mehrwert neuerer Sequenzen oder Analysemethoden wie z. B. MR-Fingerprinting oder Radiomics ist bisher noch unzureichend geklärt und somit in der aktuellen Version der Guidelines noch nicht enthalten.

Seriell durchgeführte Verlaufskontrollen im Rahmen einer Active-Surveillance-Strategie werden in der Zukunft an Relevanz gewinnen, allerdings sind die Bewertungskriterien hier noch nicht exakt definiert. So könnte z. B. der Nachweis einer *Tumorprogression* anhand einer Größenmessung auf T2w-Sequenzen oder ggf. auch durch ein Absenken des ADC-Werts im Verlauf (als Indiz für einen höheren Gleason Score) erfolgen [19]. Ähnliche Limitationen existieren auch für die Rezidivbeurteilung nach erfolgter lokaler Therapie, wobei dazu kürzlich erste Empfehlungen publiziert wurden [23].

Die Bildqualität der MRT-Sequenzen und die häufigen, meist (post)entzündlichen Veränderungen in der peripheren Zone haben ebenfalls einen großen Einfluss auf die Detektion von Läsionen [12, 13] – auch hierzu fehlt in den aktuellen Richtlinien noch eine möglichst objektive und reproduzierbare Einschätzung.

## Fazit für die Praxis

- Die PI-RADS-Richtlinien haben zur Standardisierung und damit zur Verbreitung und Akzeptanz der multiparametrischen MRT der Prostata entscheidend beigetragen.
- Die nun auf Version 2.1 aktualisierten Richtlinien stellen eine evolutionäre Verfeinerung der bestehenden Version 2.0 dar, die vor allem technische Anforderungen adressieren und Unklarheiten und Ungenauigkeiten im Scoringsystem reduzieren sollen.
- Neben der strukturierten Befundung und einem standardisierten Stichwortverzeichnis sollen diese Präzisierungen zu einer verbesserten Vergleichbarkeit zwischen Untersuchern und Zentren beitragen.
- Einige bereits aus Version 2.0 vorbekannte Limitationen bleiben jedoch weiterhin bestehen und sollten in kommenden Versionen adressiert werden.

## Abkürzungen

ADC: Apparent Diffusion Coefficient
AFMS: Anteriores fibromuskuläres Stroma
CZ: Zentrale Zone
DCE: Dynamic contrast-enhanced
DWI: Diffusionsgewichtete Bildgebung
ISUP: International Society of Urological Pathology
mpMRT: Multiparametrische MRT
PZ: Periphere Zone
PSA: Prostataspezifisches Antigen
PI-RADS: Prostate Imaging Reporting and Data System
TZ: Transitionalzone
